# The Rotating Glass Illusion: Material Appearance Is Bound to Perceived Shape and Motion

**DOI:** 10.1177/2041669518816716

**Published:** 2018-12-26

**Authors:** Hideki Tamura, Shigeki Nakauchi

**Affiliations:** Department of Computer Science and Engineering, Toyohashi University of Technology, Japan;; Japan Society for the Promotion of Science, Tokyo, Japan; Department of Computer Science and Engineering, Toyohashi University of Technology, Japan

**Keywords:** motion, object recognition, shapes/objects, surfaces/materials

## Abstract

We report a novel illusion in which a rotating transparent and refractive triangular prism (glass object) is perceived as being made of a specular reflective material (mirror), and simultaneously, its direction of rotation (clockwise or anticlockwise) is also misperceived. Our findings suggest that physical motion strongly influences viewers’ judgements of material in some situations.

Although motion aids in perception of a material and its surface properties on a rigid object (e.g., Doerschner et al., 2011; Tamura, Higashi, & Nakauchi, 2018; Ueda et al., 2015), some particular types of motion of a refractive and transparent rigid object induce mistakes in viewers’ perceptions of material and motion. We report a novel illusion in which a rotating refractive triangular prism is perceived as being of a specular reflective material and its direction of rotation is simultaneously misperceived. Figure 1(a) shows examples of the illusion (see Movie 1). A triangular prism with randomly distributed bumps, rendered using computer graphics, and rotates clockwise (when viewed from above) about the vertical axis. The left panel in Movie 1 shows this rotating object made of specular reflective material, and viewers can correctly discern its material and direction of rotation. In the right panel in Movie 1, however, viewers see a rotating object made of a transparent and refractive material, such as glass; from certain specific viewpoints, they perceive this as a specular reflective material, such as a mirror. Moreover, at that point, the object’s direction of rotation (clockwise or anticlockwise) is perceived to be reversed.

**Figure 1.**
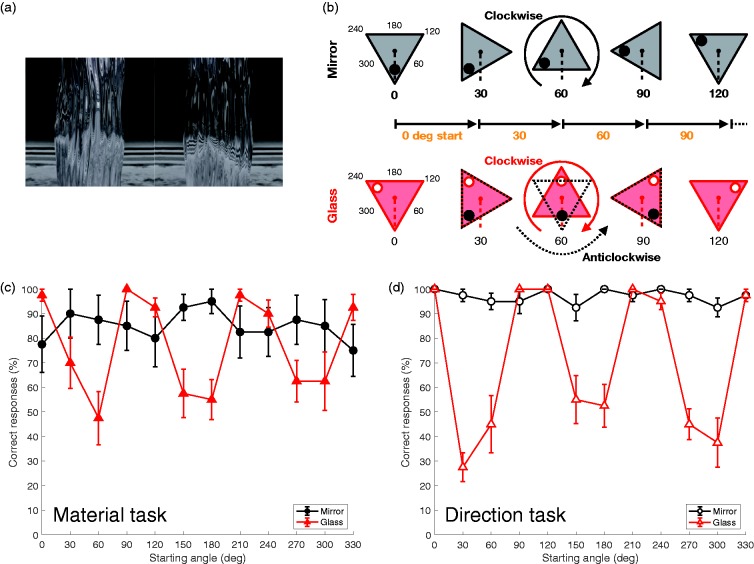
The illusion producing misperception of material and direction of rotation. (a) Example stimuli for the material and direction tasks (see also Movie 1). The left panel shows the mirror object and the right one, the glass object. (b) A diagram explaining where viewers misperceived the object. (c) Results of the material task. The horizontal axis indicates starting angles for the object’s rotation. The vertical axis indicates the percentage of correct answers. Averages across all 10 observers are shown; error bars represent the standard error of the mean. (d) Results of the direction task, presented as in (c).

**Movie 1.** (Click to play). Example stimuli (see also Figure 1(a)).

We investigated the error rate in perception of material appearance and direction of rotation. The stimuli were videos of a bumpy triangular prism rotating through 30° from 1 of 12 starting positions (see Figure 1(b)). Two versions of each stimulus were prepared in which the prism was made of different materials: “mirror,” a perfectly specular reflective surface, or “glass,” a refractive medium (with a refractive index of 1.5; its reflectance and transmittance were 0.04 and 0.96, respectively). Stimuli were rendered using a physically based renderer Mitsuba (Jakob, 2010) under realistic illumination “Uffizi Gallery” (Debevec et al., 2000). One stimulus was for a second video with 60 frame/s refresh rate and the speed of the object’s rotation was 0.5° per frame. Ten observers were exposed to the stimuli and asked to judge the material of the object (mirror or glass) in the material task. In different blocks, they were also asked to judge the object’s direction of rotation (clockwise or anticlockwise) in the direction task. The order of the two blocks was counterbalanced.

Figure 1(c) and (d) shows the percentage of correct answers of all observers in the material and direction tasks, respectively. Although performance was stable for mirror stimuli, that for glass stimuli tended to be worse at specific starting angles (30°, 60°, 150°, 180°, 270°, and 300°) in the material task (Figure 1(c)), and there was a significant difference in performance depending on the combination of material and starting angle, with a two-way repeated measures analysis of variance indicating a significant interaction, *F*(3.849, 34.645) = 9.371, *p* < .001. Similarly, performance differed at the same specific angles in the direction task (Figure 1(d)), *F*(3.278, 29.500) = 23.978, *p* < .001. These results suggest that the observers misperceived the appearance of the material and direction of rotation in both tasks, and that the starting positions in which these misperceptions occurred were consistent. Note that we present adjusted degrees of freedom using Greenhouse–Geisser correction when the criterion for the assumption of sphericity (using Mauchly’s test) was not met.

Although the object physically rotates in a fixed direction, the viewer’s visual system misperceives the object’s material and direction of rotation in certain instances, because the visual system relies on the components of motion to distinguish reflective and refractive materials (Tamura et al., 2018). For example, even if the object physically rotates clockwise, the opposite motion components anticlockwise could be dominant, depending on the complex light reflection and refraction resulting from the interactions between shape, surface properties, and illumination. This illusion suggests that these physical motions of a triangular prism with rich optical properties induce confusion in viewers’ perceptions of material and motion.

The visual system narrows down a target structure by accumulating relative motion information at a given time for the structure based on its motion (e.g., Ullman, 1979). This allows for the ambiguity to be resolved when viewing only the object’s front surface or both the front and the rear surfaces. At the specific starting angles at which the illusion occurs, a convex edge of the triangular prism made of glass was facing toward the rear (see Figure 1(b)). The visual system could be misperceiving this edge as that facing the front, as in the hollow-face illusion (Gregory, 1997; Hill & Johnston, 2007), thus reversing perception of the object’s direction of rotation. This means that the visual system more easily recognizes the object when it has a specular reflective surface and tends to ignore refractive media.

From the viewpoint of a change in material appearance, this illusion is similar to the type of illusion in which a refractive object is perceived as a specular reflective object when it is turned upside-down (Kim & Marlow, 2016). However, in that case, the authors reported on a static image illusion; the illusion we report here is a video illusion and simultaneously changes the viewer’s perceptions of material and motion. This illusion could be a new tool to further explore the relationship between material appearance and motion.
